# Cell Fate of Retinal Progenitor Cells: In Ovo UbC-StarTrack Analysis

**DOI:** 10.3390/ijms232012388

**Published:** 2022-10-16

**Authors:** Cindy L. Olmos-Carreño, María Figueres-Oñate, Gabriel E. Scicolone, Laura López-Mascaraque

**Affiliations:** 1 Instituto de Biología Celular y Neurociencias “Prof. E. De Robertis” (IBCN), CONICET and Departamento de Biología Celular, Histología, Embriología y Genética, Facultad de Medicina, Universidad de Buenos Aires, Buenos Aires 1121, Argentina; 2 Instituto Cajal-CSIC, Molecular, Cellular and Developmental Neurobiology Department, 28002 Madrid, Spain

**Keywords:** stem/progenitor retinal cells, ciliary margin, UbC-StarTrack, clonal analysis, chick embryo

## Abstract

Clonal cell analysis outlines the ontogenic potential of single progenitor cells, allowing the elucidation of the neural heterogeneity among different cell types and their lineages. In this work, we analyze the potency of retinal stem/progenitor cells through development using the chick embryo as a model. We implemented in ovo the clonal genetic tracing strategy UbC-StarTrack for tracking retinal cell lineages derived from individual progenitors of the ciliary margin at E3.5 (HH21-22). The clonal assignment of the derived-cell progeny was performed in the neural retina at E11.5-12 (HH38) through the identification of sibling cells as cells expressing the same combination of fluorophores. Moreover, cell types were assessed based on their cellular morphology and laminar location. Ciliary margin derived-cell progenies are organized in columnar associations distributed along the peripheral retina with a limited tangential dispersion. The analysis revealed that, at the early stages of development, this region harbors multipotent and committed progenitor cells.

## 1. Introduction

The chicken retina represents a good model for studying the development of the central nervous system (CNS) as well as their regeneration properties and diseases in higher vertebrates [1,2,3,4,5]. The chicken eye specification occurs at stage 9/10 of Hamburger and Hamilton (1951) (HH9/10), before the optic vesicle neuroepithelium gives rise to the retinal pigmented epithelium, neural retina and optic stalk [6]. The development of the neural retina is a conserved chronological process of cell genesis and differentiation [7,8,9]. It depends on the proliferation rate [10,11,12,13], symmetrical or asymmetrical cell divisions [14,15,16,17], progenitor cell migration [18,19], and cell differentiation [14,20,21]. Neurogenesis starts around embryonic day 2 (E2) (HH12) in the central chicken retina followed by a progressive process of cell specification and differentiation [22]. Retinal progenitor cells (RPCs) give rise to six neuronal types and one glial cell type, organized in three nuclear layers separated by two plexiform layers. Cone and rod photoreceptors are placed in the outer nuclear layer (ONL) and horizontal, bipolar and amacrine interneurons and cell bodies of radial Müller glia are located in the inner nuclear layer (INL), while projection neurons named retinal ganglion cells (RGCs) reside in the ganglion cell layer [23,24]. However, most of the major cell types could be further categorized depending on specific functions or expression of a given set of genes. A recent transcriptomic analysis of the chicken retina identified 136 cell subtypes alongside 14 developmental intermediates distributed among the retinal cell types [25]. During development, both neurons and glia are generated from RPCs, which progressively reduce their location from the neural retinal layer of the optic cup to the ciliary margin (CM). This area includes the prospective ciliary body and ciliary marginal zone (CMZ) [26,27,28]. The borderline of CM towards the retina is clearly detectable at E7, both morphologically and by the enzyme butyryl cholinesterase activity [29,30]. CMZ is clearly recognized from the ciliary body from E16 onwards [26,27] and RPCs are later confined to this area during later development and in the mature chick retina [26,27,28,31,32]. Further, according to in vitro modeling in retinospheroids, CM retinal progenitors amplify their pool throughout symmetric divisions, which increase the surface of the peripheral retina (lateralization). This is followed by asymmetric divisions and radial migration and differentiation, processes which give rise to retinal column formation in the peripheral retina [33,34]. CM progenitor cells generate all retinal cell types, but this capability decreases during development [35]. Furthermore, CM presents a transient regenerative capability during the early stages of development [36,37,38,39]. These data depict the importance of knowing the biology of CM progenitor cells.

Fate mapping analyses and gene expression showed a heterogeneous pool of RPCs in different species, such as frogs, mice or zebrafish [40,41,42]. In chickens, these RPC populations included multipotent [40,43,44,45] and fate-restricted progenitors [46,47,48,49]. Both intrinsic and extrinsic mechanisms may explain the RPC potentiality [10,50,51]. Regulatory elements have been described to be involved in the specification of fate-restricted RPCs committed to generating few retinal cell types [46,52,53,54]. Although omics studies revealed the cellular heterogeneity of adult and developmental retina, the specific temporal and topographic patterning of the generation of their different cell types from RPCs is still under study. It was recently discovered that specific temporal factors in RPCs ensured the generation of different cell types at a given time point [55]. However, those works were performed in the whole retina or in the central and peripheral retina, but the cell potency of RPCs was not focused on the CM. Therefore, lineage tracing approaches are necessary to assess the origin and fate of individual chick RPCs placed in the CM, classically assessed by viral libraries [47,56,57]. This is a crucial topic for understanding retinal development and to design successful cell retina replacement therapies. In this respect, multicolor lineage tracing methods are powerful tools for tracing clusters of cells derived from a common RPC [36,46]. In this study, we focused on the derived cell progenies of individual RPCs located in the chicken CM at early developmental time points. Specifically, CM was tracked using the genetic multicolor lineage tracing strategy named UbC-StarTrack [58]. This approach is based on the expression of six fluorescent proteins either in the cell nucleus or cytoplasm, generating a unique and inheritable barcode in multiple individual progenitors and all their derivatives. Due to its ubiquity, UbC-StarTrack has been used to unravel lineages in different mouse brain regions [59,60,61,62,63,64] as well as in cultured cancer stem cells [65]. After targeting RPCs from the CM at E3.5 (HH21-22), we deciphered the fate and clonal relationships of their derived cell progenies eight days post in ovo electroporation. Altogether, our data demonstrated that at the early stages of development, the CM harbors multipotent and committed progenitor cells that give rise to diverse retinal cell clones displaying a high level of heterogeneity. 

## 2. Results

### 2.1. Feasibility of Targeting Embryonic Chick Progenitors Using the UbC-StarTrack Approach

**Figure 1:**
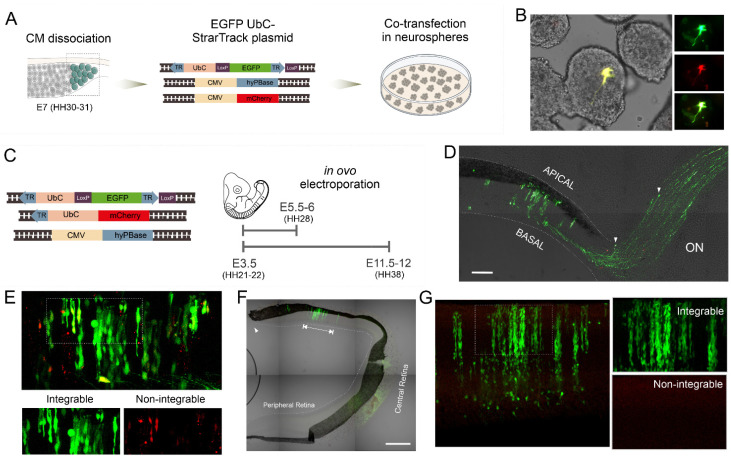
Expression of the UbC-StarTrack in vitro and in ovo. (**A**) General overview of neurospheres generation and co-transfection with an UbC-StarTrack integrable EGFP plasmid and a non-integrable vector expressing mCherry. (**B**) Epifluorescence and phase contrast merge image of co-transfected neurospheres. Positively labeled cells for both plasmids were evident in retinal neurospheres. Green (EGFP), red (mCherry) and yellow (merge). (**C**) Diagram showing procedure followed for the in ovo electroporation of an integrable UbC-StarTrack vector (expressing EGFP) along a non-integrable UbC-StarTrack vector (expressing mCherry). Labeled cells were checked either two or eight days after the IOE of the three constructs. (**D**) Epifluorescence and phase contrast merge image of a horizontal section passing throughout the ventral part of the central retina at E5.5-6 (HH28). Note the EGFP-labeled axons (arrows) heading toward the formation of the optic nerve (ON). Co-electroporation was performed in the peripheral retina and labeled cells are located next to the central retina. Scale bar 100 µm. (**E**) Labeled green (integrated), red (episomal) and yellow (both) cells are shown in short-term E5.5-6 (HH28). Inset is shown below both in green and red channels at higher magnification. (**F**) Epifluorescence and phase contrast merge image of a retinal section at E11.5-12 (HH38). The peripheral retina, which extends from the proximity of the CM (arrowhead) towards the central retina, is shown. Cell clusters are located in columns along the radial axis of the retina occupying a limited area in the peripheral retina (arrows). Nasal retina is upwards and temporal retina is downwards. Co-electroporation was performed in CM. Scale bar 500 µm. (**G**) In the long-term, E11.5-12 (HH38), just the integrated UbC-EGFP labeling persists. Merging green and red channels are successively shown.

To validate the UbC-StarTrack approach in the chicken retina, we initially tested the expression of a single StarTrack plasmid. First, neurospheres derived from the CM cells of chick retinas at E7/HH30-31 and were co-electroporated with the following constructs (Figure 1A): (1) UbC-EGFP-StarTrack, an integrable construct expressing the EGFP from the UbC-StarTrack mixture under the Ubiquitin C (UbC) promoter; (2) hyperactive piggyBac transposase (hyPBase) for gene integration; and (3) pcDNA-mCherry-LIC, a non-integrable plasmid containing mCherry, used as a control for cell transfection. Labeled cells expressing either the integrable (EGFP) or the non-integrable (mCherry) plasmids were evident in neurosphere cultures (Figure 1B), confirming the in vitro effectiveness of the UbC-StarTrack constructs in this model.

Furthermore, to assess the intensity and stable integration of the UbC-StarTrack system in the chick retina in vivo, we performed in ovo co-electroporation (IOE) with different constructs: (1) integrable EGFP-UbC-StarTrack; (2) UbC-mCherry plasmid encoding the mCherry fluorescent protein without the 3′ terminal repeat (TR) to avoid the integration by the transposase; and (3) hyPBase transposase. IOE was performed at E3.5 (HH21-22) and labeled retinal cells were examined either after two (E5.5-6; HH28) or eight (E11.5-12; HH38) days post-electroporation (dpe) (Figure 1C). At E5.5-6, labeled cells were distributed across the retinal layers forming repetitive columnar structures along the radial axis (from the apical to the basal surface) (Figure 1D). In addition, axonal bundles and some of their growth cones (arrowheads in Figure 1D) were labeled in the ventral retina, forming the optic nerve. HH21-22-derived cell progenies displayed three different patterns of expression depending on the uptake of one vector (integrable UbC-EGFP-StarTrack or non-integrable UbC-mCherry plasmid) or both (Figure 1E) two dpe. To assess the UbC-StarTrack plasmid integration in relation to the dilution of non-integrated plasmids, retinas were analyzed eight dpe (Figure 1F). Horizontal sections of the retina exhibited the CM (arrowheads in Figure 1F), the peripheral retina bordering the CM and the central retina (Figure 1F). EGFP-labeled cells were grouped into columnar structures extending up to 1000 μm (arrow in Figure 1F). At E11.5-12, the number of red positive cells expressing the non-integrated sequence were scarce, whereas green cells corresponding to the UbC-EGFP-StarTrack integrated construct were brightly expressed forming retinal columns (Figure 1G). Thus, the UbC-EGFP-StarTrack was stably integrated by the hyPBase in chick RPCs and inherited by their derived cell progenies. Moreover, UbC-StarTrack was suitable for the morphological identification of retinal cell types derived from RPCs, allowing the determination of their topographic organization along the radial and tangential axes of the retina.

### 2.2. Targeting Single Progenitors in the Chick Retina

**Figure 2:**
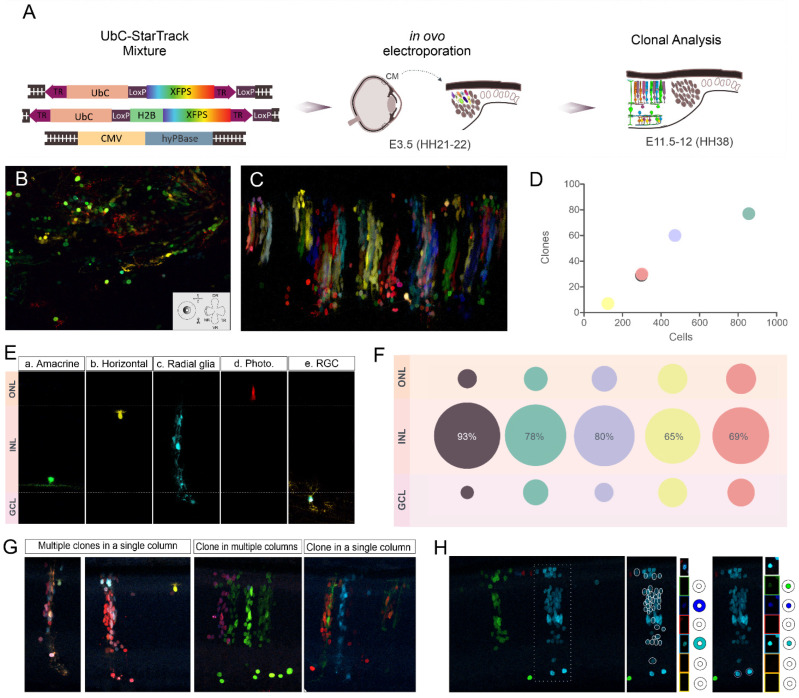
In ovo electroporation of the UbC-StarTrack. (**A**) General overview of IOE of UbC-StarTrack into the CM of chick embryos at E3.5 (HH21-22) and their analysis at E11.5-12 (HH38). (**B**,**C**) Confocal merged images of retinal whole mount (**B**) and retinal horizontal section (**C**) from chick embryos at E11.5-12 (HH38) after IOE of UbC-StarTrack at E3.5 (HH21-22). (**B**) Cell clones are observed from the external surface of the retina and individual cells are visualized from a perpendicular or oblique incidence in whole mounts. (**C**) Labeled cells observed from a longitudinal incidence, form columnar associations between the internal (basal border) and external (apical border) limits of the neural retina in horizontal sections. UbC-StarTrack labels cells in both the cytoplasm and nucleus, allowing the cells somas and processes to be seen clearly. (**D**) Graph representing the relationship between the number of clones and the number of cells detected in each of the five analyzed chick embryos. (**E**) Confocal images showing the characteristic morphology of chicken retinal cells in horizontal sections at E11.5-12 (HH38) after E3.5 (HH21-22) IOE with UbC-StarTrack. A labeled amacrine cell (a), a horizontal cell (b), a radial glia (c), a photoreceptor (cone) (d) and a retinal ganglion cell (RGC) (e) are shown. (**F**) Percentages of labeled cells located in the ONL, INL and CGL in each of the five chicken embryos analyzed. (**G**) Confocal merge images of all XFPs showing examples of sibling cells arranged in columns in the embryo retina. (**H**) Confocal image merge of all XFPs is shown on the left side. The inset shows an apparent homogenous clone in a single column. However, when analyzing the fluorescent expression at each confocal channel separately it is shown that the column is formed by two different clones: (1) a trilaminar clone formed by cells expressing mCerulean and mT-Sapphire in the cytoplasm (middle) and (2) a monolaminar (GCL) clone formed by cells expressing mCerulean, mT-Sapphire and EGFP in the nucleus (right).

Once the stability and integration of the UbC-StarTrack in the chick retina was probed, the complete plasmid StarTrack mixture (Figure 2A) was electroporated into the CM at E3.5 (HH21-22). Cell-derived progenies were brightly and stably labeled by XFPs both in the cytoplasm and cell nucleus eight dpe at E11.5-12 (HH38, Figure 2B,C). The cell morphology and the topographic organization of labeled cells were not clearly appreciated in the whole retina mount (Figure 2B). However, in retinal horizontal sections, groups of labeled cells were organized into radial columns along the peripheral neural retina (Figure 2C). This columnar organization along the radial axis of the retina enabled a better characterization of cell morphology, cell relationships and laminar location. Therefore, clonal analysis was performed in consecutive retinal horizontal sections. Cell segmentation and fluorescent reporter expression was determined at each labeled retinal cell using a pseudo-automated pipeline in ImageJ in five electroporated embryos. Color-codes were determined by the expression, location and intensity of the six different XFP expressed in both nucleus or cytoplasm [47]. Sibling cells were those cells gathered by the same color-code. A total of 2050 UbC-StarTrack labeled cells were grouped into 203 clones. Although the number of labeled cells varied in individual embryos, the number of clones correlated to sample size, indicating a link between sample size and the number of targeted single progenitors (Figure 2D). Furthermore, the cell type identity of UbC-StarTrack-tracked cells was revealed by the shape of both cell bodies and processes, and the laminar disposition along the radial axis of the retina was found at this developmental time point (Figure 2E). The somas of amacrine cells (Figure 2E(a)) were located on the inner boundary of the INL with horizontal dendrites extended up to the inner plexiform layer (IPL). The horizontal cell bodies (Figure 2E(b)), situated at the outer boundary of the INL, displayed dendrites extending into the outer plexiform layer (OPL). The radial glial cells (Figure 2E(c)) spanned along the entire neural retina with their cell bodies found at different levels of the INL. Photoreceptors (Figure 2E(d)) displayed cylindrical shape bodies with their nuclei positioned in the outer nuclear layer (ONL). Based on the literature, there are no rods at the analyzed time point (HH38) in the chick retinas. Thus, all cells in the ONL with photoreceptor morphology are considered as a cone photoreceptor cell type. Large RGCs somas were placed in the GCL with a dendritic tree extending into the INL and an axon that may extend throughout the layer of optical fibers (Figure 2E(e)). Although it is possible to find displaced amacrine cells in the GCL, ganglion cells have a large soma that allowed us to morphologically differentiate them from amacrine cells. However, cells with no clear characterization were not included in the study. To better classify the tracked cell lineages, analyzed cells were sorted according to their laminar location in the ONL, INL or GCL (Figure 2F). Most labeled cells were located in the INL (78.83%, with a range of 92.95–65.32% at different replicates), followed by photoreceptors in the ONL (11.32 %, with a range per sample of 5.03–17.74%) and RGCs (9.85%, within the range of 2.01–16.94% at different replicates) in the GCL (Figure 2F). As described above, different cell types were contained in these three layers at the time of the analysis: ONL displayed labeled cone photoreceptors (232 cells) and the RGCs (202 cells) were located in the GCL. However, labeled cells of the INL (1616 cells) encompassed interneurons (horizontal and amacrine cells), as well as radial glial cells and other undifferentiated and migratory cells with somas sited into the INL.

Labeled cells organized into compartmentalized columns in the retinal horizontal sections (Figure 2C,G). Sibling retinal cells arranged along with either one, two or more labeled cellular columns. Clones, identified by sibling cells with the same color-barcode, are distributed as follows: (1) cells from different clones intermixed in a single column (Figure 2G, multiple clones into a single column), (2) clones with sibling cells dispersed in multiple columns (Figure 2G, clone in multiple columns) and (3) a clone in a single column (Figure 2G). It is important to note that cell groups, apparently related by their color in the merged image, may not be clonally related when analyzing their fluorescent barcode expression at each separated channel (Figure 2H). 

### 2.3. Clonal Arrangements in the Retina after UbC-StarTrack Targeting

**Figure 3:**
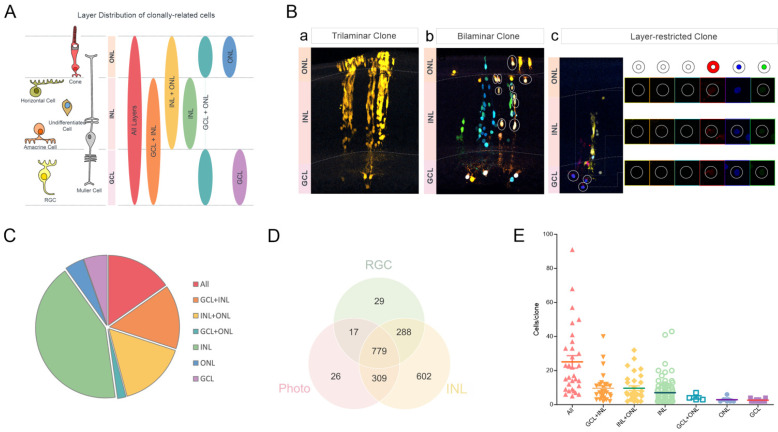
Retinal layer distribution and the clonal size of cell-derived progeny after targeting CM progenitors. (**A**) General overview of layer distribution of clonally related cells. Retinal cell types are represented on the left side, whereas the seven group classifications depending on the clone layer distribution are represented on the right side. (**B**) Merge the confocal images of a trilaminar clone (a) with cells located at different retinal layers (ONL, INL and GCL), a bilaminar clone (b) formed by a photoreceptor, three horizontal cells and six undefined cells and a layer-restricted clone (c) formed by three RGCs. Circles represent cells belonging to the same clone. Detailed expression of XPF defining the color code of sibling cells as well as the schematic representations are shown in (c). (**C**) Pie chart displaying the proportions of the different layer distribution of sibling cells. (**D**) Venn diagram representing the number of cells belonging to clones formed by different combinations of cell types, such as RGCs, photoreceptors and INL cells. (**E**) Size of the clones distributed along the different retinal layers. Clones containing cells at all three layers were the larger ones. GCL: ganglion cell layer; INL: inner nuclear layer; ONL: outer nuclear layer; RGC: Retinal ganglion cell; Photo: Photoreceptor (cone).

To further investigate the cell composition among examined clones, seven types of clones were determined according to the distribution of their sibling cells throughout the different layers of the chick retina (Figure 3A). Thus, depending on the layer distribution, clones were classified in trilaminar, bilaminar or one layer-restricted clones (Figure 3B). Trilaminar clones were distributed throughout the GCL, INL and ONL (15.3%). Bilaminar clones displayed distinct layer arrangements with sibling cells located at the GCL and INL (14.8%), the INL and ONL (15.8%) or the GCL and ONL layer (2%). Layer-restricted clones were confined to either the INL (42.4%), ONL (4.4%) or GCL (5.4%) (Figure 3C). Out of a total of 2050 cells, only 26 cells were included in clones confined to the ONL, 29 cells in the clones were restricted to the GCL and 17 cells in bilaminar clones were limited to both ONL and GCL (Figure 3D). Most analyzed cells belonged to the clones widespread across all three layers (779 cells) and limited to INL (602 cells) (Figure 3D). Regarding clonal size, the biggest clones were the trilaminar ones (25.1 ± 3.7 cells per clone) (Figure 3E), while the smallest clones were formed by RGCs (2.6 ± 0.2 cells per clone), followed by photoreceptor clones (2.9 ± 0.4 cells per clone). 

### 2.4. Lineage Specification of Retinal Progenitors

**Figure 4:**
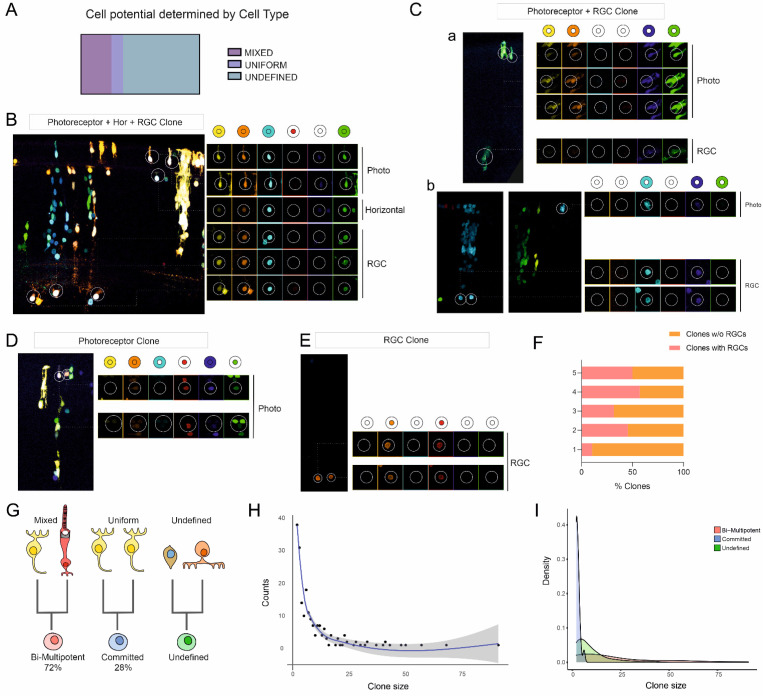
Progenitor potential and clonal heterogeneity deciphered using UbC-StarTrack. (**A**) Schematic representation of the proportions of clones according to their cell type composition: mixed clones included at least two differentiated cell types, uniform clones only one and undefined clones were composed by cells for which an identity could not be designated. (**B**) A mixed clone was formed through three differentiated cell types: two photoreceptors, a horizontal cell and three RGCs. (**C**) Examples of mixed clones formed by both photoreceptors and RGC. Sibling cells could appear in the same horizontal retinal slice (a) or in adjacent ones (b). (**D**,**E**) Uniform clones were those composed by only one differentiated cell type, as photoreceptors (**D**) or RGCs (**E**). Cells from each clone are circled at each image. For all cells, the details of the XFPs expression is shown. Clone color codes are represented by the schematics. (**F**) Proportion of the clones that included RGCs in the five analyzed replicates. (**G**) Schematics displaying the progenitor potential deciphered by the clone composition. Mixed clones were derived from bi- or multipotent progenitors, whereas uniform clones arose from committed progenitors at E3.5 (HH21-22). There was a proportion of clones for which progenitor potential was not assessed due to the presence of sibling cells within the clone for which cell identity could not be found at the time of the analysis at E11.5-12 (HH38). (**H**) Scatterplot showing the accumulation of clones, named counts, of a determined size. Blue line shows the predicted fitting by local polynomial regression, and the grey shadow represents the standard error of the predicted model when using a 0.95 confidence interval. (**I**) Density chart comparing the distribution of the clonal sizes at E11.5-12, depending on the nature of the E3.5 RPCs: bi-multipotent, committed or undefined.

We next sought to explore the cell potential of StarTrack-targeted progenitors through a retrospective clonal analysis, comparing the distinct or shared color barcodes of targeted cells. We considered four differentiated cell types recognized by morphology at E11.5-12 (HH38) that included cone photoreceptors, horizontal cells, amacrine cells and RGCs. Moreover, those cells in the INL that could not be identified by their morphologies were sorted as undefined cells, which may include undifferentiated or immature cells, including migrating and non-migrating cells. The potentiality of RPCs was retrospectively deciphered by the cell types forming each individual clone (Figure 4A). Mixed clones were those named as bilaminar or trilaminar clones, composed of two or more differentiated retinal sibling cells that arose from a common bipotent/multipotent progenitor at the time of the analysis. Some progenitors were clearly defined as multipotent when presenting at least three differentiated retinal cell types (Figure 4B) or bipotent when presenting two, such as photoreceptors and RGCs (Figure 4C). Sibling cells could appear in the same retinal horizontal (Figure 4C(a)) or in adjacent sections (Figure 4C(b)). However, mixed clones arising from bi-multipotent RPCs could also contain undefined cells, alongside at least two differentiated cells at different combinations, such as photoreceptors and horizontal cells (Figure 3B(b), bilaminar clone). Uniform clones encompassed layer-restricted clones with a single cell type derived from a committed progenitor at the time of the study. Uniform clones were formed by photoreceptors (Figure 4D) or RGCs (Figure 4E). On the other hand, 34.5% of the labeled progenitors gave rise to clones containing RGCs, which are the first cell type to differentiate (Figure 4F). However, at the moment of the analysis, it was not possible to determine potentiality in the 64.53% of targeted RPCs as their progeny consisted of cells whose identity could not be established by morphology. Undetermined clones could include one differentiated cell type, but as the further fate of undefined cells could not be deciphered, we concluded that the potentiality of these progenitors remained unknown at the time of the analysis. Thus, clones with identifiable cell types originated from the 35% of targeted progenitors, bipotent/multipotent RPCs being the most common (72.22%) (Figure 4G). Similarly, 9.85% of the derived clones after targeting progenitors at HH21-22 with UbC-StarTrack arose from committed RPCs at the time of the analysis. The proliferative capacity of targeted single RPCs was checked by comparing the distribution of the accumulated counts for clone sizes (Figure 4H). Clones with up to 12 cells fitted better in a local polynomial regression model with minimum standard error. To assess whether differences on proliferative capacity was a feature related to lineage, clone size densities were plotted, segregating RPCs based on their potency (Figure 4I). The examination of the distribution of densities revealed differences on the proliferative rates of targeted progenitors regarding their fate. Altogether, these findings indicate that the RPCs have different cell potencies at early developmental times, confirming the heterogeneity of this cell population.

## 3. Discussion

Our data revealed that at the early stages of retinal development, the ciliary margin harbors multipotent and committed progenitor cells that give rise to retinal cell clones displaying a high level of heterogeneity. Sibling cells assembled in a columnar cluster arrangements along the radial axis of the peripheral retina formed mixed or uniform clones depending on both cell type composition and layer location. In addition, UbC-StarTrack [58] ensured the tracking of the cell-derived progenies at E11.5-12 (HH38) of single RPCs targeted at E3.5 (HH21-22). This would not be possible without plasmid integration into the genome of transfected cells [58,66,67]. In avian species (chicken embryos) the piggyBac system has been proved to mediate the insertion of a transgene during developmental stages [68,69,70,71]. The persistence of the integrable UbC-EGFP plasmid expression and the loss of the non-integrable UbC-mCherry eight days after IOE, evidenced the effectiveness of the genomic integration of the UbC-StarTrack system, as previously reported in mice [67]. These features allowed us to overcome the previously described limitations of other methods for clones and lineage tracking [72].

The accurate identification of chick retinal cells at HH38, based on cellular morphology and laminar location, has been confirmed by molecular ratification [20,22,47]. Neuroepithelial/progenitor cells, migratory cells and the retinal cell types characterized by their location within the different retinal layers and distinctive morphological characteristics are compatible with those previously reported [20,73].

### 3.1. Distribution of Cellular Progenies in Retina after Targeting Single Progenitors in the Ciliary Margin

The ciliary margin is a developing retinal area with a transitory capability to regenerate a chicken retina [3,36,37,38]. Most omic studies focused on the central retina without differentiating the specific features of the peripheral retina or the CM. In this in ovo lineage tracing, we specifically targeted single progenitors sited in CM at E3.5 (HH21-22), revealing the cell dispersion pattern and identity of each sibling labeled cell progenies at E11.5-12 (HH38) along the tangential retinal axis. Thus, CM progenitors gave rise to the peripheral chicken retina, the earliest part of the retina specialized for specific functions different from the central retina [22,26]. 

The space observed between the CM and the labeled clones in the peripheral retina at E11.5-12 (HH38) demonstrates the process of non-labeled RPCs lateral displacement throughout symmetric cell divisions, which expand the surface of the peripheral retina. This cellular process was defined as lateralization in an in vitro model developed in retinospheroids [34,74]. Furthermore, the asymmetric cell divisions of RPCs, followed by cell migration and differentiation, give rise to the columnar organization of clones and the retinal lamination, processes named radialization and lamination [34,74]. Regarding these processes, we showed that RPCs of the CM gave rise to the different proportions of cell types in the three retinal layers. Previous data in the whole retina using scRNA-seq reported that the percentage of photoreceptors increases at later stages of development [25], similar to what we found at E11.5-12. According to the declining center-peripheral developmental gradient, we detected a slightly higher percentage of INL cells (77.2%), including undifferentiated cells, than the 64% reported in the central retina [20].

The columnar arrangement displayed by the labeled cell progenies along the retinal radial axis (apical–basal) and the limited tangential cell dispersion along the anterior–posterior axis indicates the limited level of the symmetric cell divisions of targeted RPCs and the migratory paths of progenies, showing a predominant process of radial migration and a limited extension of tangential migration. Thus, clonally related neuroblasts aligned on the radial axis of the retina, while differentiated retinal cells are displaced tangentially [18,75]. These results support previous data that provide in vitro evidence that radial glia processes stabilize cells within columns, limiting the tangential migration of cells belonging to columnar organized clones [74]. 

### 3.2. Heterogeneity of Ciliary Margin Progenitors Using UbC-StarTrack

StarTrack provides progenitor potentiality by a retrospective analysis of the cell-derived progeny of individual targeted progenitors at specific developmental time points. At E11.5-12 (HH38), clonal analysis allowed the identification of differentiated retinal cells generated at that developmental time point, corresponding to the early phase of neurogenesis [72]. RGCs are the first neural type generated in the retina across development, being classified as different subtypes regarding the expression of a combination of transcription factors [24]. Photoreceptor percentages vary with species, being a majority in the chick retina cones as opposed to rods [20]. At the time point of the analysis there were no rod photoreceptors reported in the peripheral chicken retina, so we only refer to cones. Even though cones may be subclassified intp four different subtypes regarding their UV sensitivity [42], we refer to them as the same cell type due to the impossibility of a further classification when using StarTrack analysis. In fact, RGCs and cones photoreceptors were the two more frequently differentiated cells in the peripheral retinas analyzed, while an important proportion of undefined labeled cells were found in the INL. Regarding the interneurons in the INL, only horizontal and amacrine cell types were included due to the fact that bipolar cells differentiate later in development at the peripheral retina, and therefore they are not recognizable by morphology when performing the electroporation of CM RPCs at E3.5 (HH21-22) and analysis at E11.5-12 (HH38). 

At the early stages of development (HH21-22) the CM harbors a heterogeneous population of RPCs, including multipotent, bipotent and committed progenitors. Mixed clones, composed of two or more cell types, originate either from VSX2 +/LHX2+/PAX6+ multipotent progenitors in chickens [46] or RPCs characterized by Atoh 7 expression [47,72]. Regarding CM RPCs, they constitute a heterogeneous progenitor population with different cell potential or at least with different temporal patterns of lineage specification. According to this last possibility, progenitor potential could be differentially modified at distinct time points throughout development depending on their gene expression [51,56,72]. 

In addition, 34.5% of the cell progeny derived from E3.5 CM-targeted RPCs, generated clones without RGCs, which are derived either from RPCs expressing ASL1, which rarely produces RGCs [53], or other fate-restricted RPC populations [48,76,77]. We also detected mixed clones formed by cells located at different layers, such as those formed by RGCs and photoreceptors. Uniform clones (cone photoreceptors or RGCs) revealed the presence of specified RPCs at the time of the analysis, suggesting a short expanding process before the last symmetric division. Our results showed a high level of proliferative capacity diversity in the RPCs located in a specific area, such as the CM. Moreover, clones detected after StarTrack targeting revealed a larger diversity in cell composition and smaller clonal size than those reported after retroviral infections [43], most likely due to the increment of the combinatorial events in a single progenitor. Huge variability in the fate of RPCs could be explained by their different proliferative rates [40], whereas some studies endorse the idea of a population of equal potentiality and a stochastic determination of their cell fate [51,78]. Accordingly, our results provided data supporting both the cell heterogeneity of retinal clones as well as the lineage potency of embryonic RPCs.

Altogether, at the early stages of chick development, the CM harbors multipotent, bipotent and committed progenitor cells. This reveals the heterogeneity of the RPCs, which gives rise to cell clones arranged in columns along the radial axis of the retina. Furthermore, clones formed from CM RPCs are smaller and more heterogeneous structures than those previously described in other retinal areas. These findings are important for building a broader map for understanding the process of retinal development, which is useful when designing regeneration therapies based on cell transplantation.

## 4. Materials and Methods

### 4.1. Animals

Fertilized Cornish chicken eggs (Santa Isabel Farm, Cordoba, Spain and Rosenbush Institute, Buenos Aires, Argentina) were incubated at 38 °C and 60% humidity and staged according to Hamburger and Hamilton stages (HH) [79]. In ovo electroporations (IOE) were performed at 88 h of incubation. Due to the fast growth occurring at early developmental time points, similar incubation times can include different HH stages. Thus, the developmental stage of the embryos was determined considering the morphological characteristics of the eye (pigmentation) and the limbs, as described by the Atlas of Chick Development [80]. Embryos included for IOE corresponded to HH21-22, matching 3.5–4 incubation days. Embryos were collected at E5.5-6 (HH28) and E11.5-12 (HH38) after IOE and at E7 (HH30-31) for neurosphere cultures and transfections. 

### 4.2. Plasmids

UbC-StarTrack [58] is a mixture of twelve piggyBac transposon constructs encoding six fluorescent proteins expressed in the nucleus or cytoplasm and driven by a ubiquitous promoter (UbC). The hyperactive piggyBac transposase (hyPBase), driven by a CMV promoter, allows the integration of the plasmid into the transfected cells. The fluorescent proteins are the yellow fluorescent protein (YFP), monomeric Kusabira Orange (mKO), mCerulean, mCherry, mT-Sapphire and enhanced green fluorescent protein (EGFP). To evaluate the efficiency of this system in the chicken model, we also used, as a transfection control, a ubiquitous non-integrable plasmid containing mCherry (pcDNA-mCherry-LIC, Addgene, Watertown, MA, USA, Plasmid #30125). 

### 4.3. Neurospheres Transfection

Chick embryos were removed from the eggs at E7 (HH30-31) and then decapitated. The cornea, lens and vitreous humor were removed and discarded. The eye was extended and separated into 4 regions (nasal, dorsal, temporal and ventral), based on the location of the choroidal fissure. CM and neural retina (NR) were separated from retinal pigmented epithelium (RPE). CM was divided into small sections, which were mechanically dissociated. Dissociated cells were resuspended and cultured in float in a cell culture flask with 75% Dulbecco’s Modified Eagle’s Medium (DMEM), 25% DMEM/F-12 (Invitrogen), epidermal growth factor (EGF, 20 ng/mL) (PeproTech, Cranbury, NJ, USA), fibroblast growth factor -2 (FGF-2, 20 ng/mL) (Sigma, St. Louis, MO, USA), B27 supplement 1% (Gibco, Cranbury, NJ, USA) and an antibiotic (penicillin–streptomycin) of 0.5% (Invitrogen, Cranbury, NJ, USA); at 37 °C with 5% CO_2_ for 3 days [81]. Then, neurospheres were seeded into 24-well plates with the same culture medium (100 μL per well). Next, 0.5 μg of each plasmid, pcDNA3 mCherry LIC vector (Addgene, Watertown, MA, USA, Plasmid #30125), pPB-UbC-EGFP (UbC-StarTrack), 1 μg of hyPBase and 6 μL of Lipofectamine 2000 (Invitrogen) were mixed in 200 μL of the same culture medium, added to the neurospheres and incubated at 37 ° C for 2 h. Then, the culture medium was removed from each well and the neurospheres were resuspended and cultured in a new well containing the culture medium with an antibiotic at 37 °C in a humid atmosphere with 5% CO_2_. Incubation times, volumes and concentrations used for both the transfection reagent and the plasmids were followed according to the manufacturer’s lipofection protocol (Invitrogen, Cranbury, NJ, USA). After 30 days, the neurospheres were fixed with 4% paraformaldehyde (PFA)-PBS for 30 min at room temperature (RT).

### 4.4. In Ovo Electroporation (IOE)

The electroporation of CM was performed at E3.5 (HH21-22), using a modification of the technique previously described by Doh et al., 2010, and Islam et al., 2012. Microinjections were performed using pulled glass capillaries. A mixture of the 12 plasmids (with 2 μg/μL of DNA) plus the hyPBase and 0.025% Fast Green dye (Sigma) was injected into the intravitreal space (between the vitreous humor and the neural retina) by moving from the caudal to rostral direction towards the beak. The injection site was placed along the dorsal region of the eye, opposite the main bundle of blood vessels entering the eye from behind the head towards the peak tangential to the surface of the retina. Every IOE was performed to consistently target the same injection site in each embryo to minimize variation. Thereafter, a platinum-tipped, custom-made electrode (anode) was placed above the head of the embryo and on the surface of the albumin, while the negative electrode (cathode) was located below the spine at a deeper point than the anode so that the eye was situated between the electrodes. Five pulses of 15 mV of 50 ms length and at 950 ms intervals were applied using aMLab2009 electroporator. Then, the window on the shell was sealed and eggs were returned to the incubator until collected for analysis (2 or 8 days after IOE).

### 4.5. Tissue Processing

Embryos were collected 2 (E5.5/E6 (HH28)) and 8 (E11.5/E12 (HH38)) days after IOE. Heads were fixed in 4% paraformaldehyde (PFA) in PBS at 4 °C for 24 h and then stored in PBS at 4 °C until processing. The electroporated eyes were dissected, embedded in agar and vibratome sectioned at 50 µm. Sections were horizontally oriented along the naso-temporal retinal axis and mounted with Mowiol.

### 4.6. Image Processing and Data Analyses

Neurospheres were observed under an IX81 Olympus inverted microscope with phase-contrast and epifluorescence. Fluorescent labeling in both embryonic sections and whole mounts was visualized under an epifluorescence microscope (Nikon, Eclipse E600) with the appropriate filter cubes previously described by Figueres-Oñate et al., 2016. Images were acquired using a Leica SPS8 confocal microscope and further analyzed with a custom macro integrated into ImageJ Version-1.53c (NIH) [58]. The first step in the macro analysis was to appropriately select labeled cells for each image in the different confocal channels in order to make a positive cell selection for each fluorescent protein (XFP). Subsequently, the data were automatically organized in a table, designating each labeled cell a specific color code. Combinations were organized according to a code of six numbers, where 0 indicates the absence of fluorophore and 1–6 indicate the presence of different XFP. All images of retinal sections with labeled cells from each animal were evaluated.

Statistical data, collected from five embryos, were obtained using Prism 5 (GraphPad, San Diego, CA, USA). A total of 203 clones were rearranged after analyzing 2123 cells from all five embryos. Data are displayed through the manuscript as the mean plus the standard error of the mean (SEM). Plots and graphs were obtained using R-studio and Prism 5 (GraphPad). Graphical images were created with Adobe CS5 (InDesign and Illustrator).

## Data Availability

Not applicable.
